# Effect of Speech Rate on Neural Tracking of Speech

**DOI:** 10.3389/fpsyg.2019.00449

**Published:** 2019-03-08

**Authors:** Jana Annina Müller, Dorothea Wendt, Birger Kollmeier, Stefan Debener, Thomas Brand

**Affiliations:** ^1^Cluster of Excellence ‘Hearing4all’, Carl von Ossietzky Universität Oldenburg, Oldenburg, Germany; ^2^Medizinische Physik, Department of Medical Physics and Acoustics, Carl von Ossietzky Universität Oldenburg, Oldenburg, Germany; ^3^Hearing Systems, Hearing Systems Group, Department of Electrical Engineering, Technical University of Denmark, Kongens Lyngby, Denmark; ^4^Eriksholm Research Centre, Snekkersten, Denmark; ^5^Neuropsychology Lab, Department of Psychology, Carl von Ossietzky Universität Oldenburg, Oldenburg, Germany

**Keywords:** listening effort, neural tracking of speech, linguistic complexity, speech rate, time-compressed sentences, time-expanded sentences, pupillometry, speech comprehension

## Abstract

Speech comprehension requires effort in demanding listening situations. Selective attention may be required for focusing on a specific talker in a multi-talker environment, may enhance effort by requiring additional cognitive resources, and is known to enhance the neural representation of the attended talker in the listener’s neural response. The aim of the study was to investigate the relation of listening effort, as quantified by subjective effort ratings and pupil dilation, and neural speech tracking during sentence recognition. Task demands were varied using sentences with varying levels of linguistic complexity and using two different speech rates in a picture-matching paradigm with 20 normal-hearing listeners. The participants’ task was to match the acoustically presented sentence with a picture presented before the acoustic stimulus. Afterwards they rated their perceived effort on a categorical effort scale. During each trial, pupil dilation (as an indicator of listening effort) and electroencephalogram (as an indicator of neural speech tracking) were recorded. Neither measure was significantly affected by linguistic complexity. However, speech rate showed a strong influence on subjectively rated effort, pupil dilation, and neural tracking. The neural tracking analysis revealed a shorter latency for faster sentences, which may reflect a neural adaptation to the rate of the input. No relation was found between neural tracking and listening effort, even though both measures were clearly influenced by speech rate. This is probably due to factors that influence both measures differently. Consequently, the amount of listening effort is not clearly represented in the neural tracking.

## Introduction

Speech comprehension in difficult listening environments can be very demanding and effortful. Thus, the necessity to selectively steer attention may require more cognitive resources and therefore enhance listening effort. Factors such as attention not only influence listening effort, but also result in a stronger representation of the attended speech in the listener’s neural response (e.g., Mesgarani and Chang, 2012; O’Sullivan et al., 2014). This connection leads to the question of how close the amount of listening effort is reflected in the neural response. Therefore, the aim of the current study was to systematically manipulate task demands via linguistic complexity and speech rate and to investigate the influence of these two different manipulations on listening effort as quantified by subjective ratings and pupillometry and on the neural response in difficult multi-talker situations.

Listening effort has been of increasing research interest and has been investigated using different techniques (e.g., Rudner et al., 2012; McGarrigle et al., 2014). Perceived effort can be measured by self-reporting, captured by means of questionnaires, or rating scales (e.g., Gatehouse and Noble, 2004; Krueger et al., 2017). On the other hand, pupillometry as a physiological measure has long been known to reflect effort (Hess and Polt, 1964; Kahneman and Beatty, 1966); in this measure, changes in pupil dilation, controlled by the sympathetic nervous system, are recorded (Sirois and Brisson, 2014; Schmidtke, 2017 for a review). Many recent studies investigated listening effort for different listening situations using pupillometry (e.g., Kuchinsky et al., 2013; Zekveld et al., 2014; Koelewijn et al., 2015; Wendt et al., 2016). Increasing background noise and decreasing intelligibility result in an increase in pupil dilation, indicating greater listening effort (Zekveld et al., 2010, 2011). However, this is only the case until a certain point: recent studies show signs that listeners “give up” at performance levels below 50% correct recognition, i.e., the peak pupil dilation decreases at low performance rates (Wendt et al., 2018). Listening to speech masked by a single talker requires more effort than listening to speech masked by stationary noise (Koelewijn et al., 2012). Ohlenforst et al. (2017) investigated in a comprehensive review whether listening effort is increased for hearing-impaired listeners compared to normal-hearing listeners. They could show that hearing impairment increases listening effort but only when effort is captured with the physiological measure of EEG and not with subjective or behavioral measures. Furthermore, the review shows a lack of consistency and standardization across studies that measured listening effort.

The neural response of a listener can phase-lock to the slow-amplitude modulations of a speech stream (e.g., Ahissar et al., 2001) which is called neural entrainment. Neural entrainment is modulated by high-level processes such as attention and prediction, so that high excitability phases of neural oscillations align to important events of the acoustic input (Schroeder and Lakatos, 2009). Kösem et al. (2018) demonstrated that neural entrainment persists after the end of a rhythmic presentation and that the entrained rate influences the perception of a target word. Thus, neural oscillations shape speech perception (e.g., Bosker and Ghitza, 2018). Many studies demonstrated that selective attention modulates neural entrainment and leads to a selectively enhanced representation of the attended stream (e.g., O’Sullivan et al., 2014; Mirkovic et al., 2015, 2016; Petersen et al., 2017; Müller et al., 2018). For instance, Petersen et al. (2017) presented continuous speech either in quiet or masked by a competing talker at different signal-to-noise ratios (SNRs) to participants with hearing impairment and investigated the influence of hearing loss, SNR, and attention on neural tracking of speech. Neural tracking of speech is the phase-locked neural response to the attended speech calculated as the cross-correlation between the speech-onset envelope (SOE) and the electroencephalogram (EEG) of the listener. Amplitude and latencies of the resulting cross-correlation components corresponding to the auditory evoked potentials (Horton et al., 2013) are denoted P1_crosscorr_, N1_crosscorr_, and P2_crosscorr_ (adopted from Petersen et al., 2017). Greater hearing loss resulted in a smaller difference in neural tracking between attended and ignored speech. Furthermore, Petersen et al. (2017) reported a reduced amplitude of neural tracking for lower SNRs as well as for the ignored speech compared to the attended speech. The contributions of acoustic properties and cognitive control on neural tracking of speech are not fully investigated (Wöstmann et al., 2016). Therefore, the question arises whether neural tracking is only sensitive to changes in the acoustics (such as SNR) and to attentional influences, or whether it is also affected by the amount listening effort a participant experienced not caused by attentional influences.

In order to answer the aforementioned question, we varied task demands during a speech comprehension task by varying linguistic complexity and speech rate of sentences. Pupillometry and a categorical rating scale were applied to obtain two different indicators of listening effort. EEG was applied to record neural tracking of speech. The variation of linguistic complexity was achieved using the Oldenburg Linguistically and Audiologically Controlled Sentences (OLACS), which include seven sentence structures that differ in their level of linguistic complexity (Uslar et al., 2013) and linguistic processing. The speech rate of OLACS was expanded and compressed to a 25% slower and a 25% faster version which influences the signal properties. The reason for time-expansion and time-compression is to have two speech rates that clearly differ from each other to receive large differences in task demands. The variations in task demands in combination with recordings of listening effort and neural tracking allowed us to investigate the following five hypotheses.

Previous studies showed a small influence of linguistic complexity on speech intelligibility (Uslar et al., 2013) and on listening effort, with larger effort for more complex sentence structures (Piquado et al., 2010; Wendt et al., 2016). Based on these studies, our first hypothesis (H1) was that listening effort and speech intelligibility are influenced by the level of linguistic complexity: higher complexity leads to higher listening effort (quantified by effort rating and pupillometry) and higher speech reception thresholds (SRTs).

Furthermore, Wingfield et al. (2006) reported that syntactic complexity reduces speech comprehension, especially for hearing-impaired and older listeners and that this effect is increased for time-compressed sentences. Further studies showed that speech comprehension is decreased for time-compressed, faster sentences (e.g., Versfeld and Dreschler, 2002; Peelle and Wingfield, 2005; Ghitza, 2014; Schlueter et al., 2014), whereas, time-expanded, slower sentences did not influence speech recognition performance (Korabic et al., 1978; Gordon-Salant and Fitzgibbons, 1997). Zhang (2017) investigated the impact of task demand and reward on listening effort quantified by pupillary data using five different speech rates and demonstrated that effort was influenced by both. Since there is a relation between speech intelligibility and listening effort and the former is affected by speech rate, we also expected speech rate to influence the latter. Moreover, the study by Zhang (2017) showed an impact of speech rate on pupillary data, with larger peak-pupil dilations for faster speech than for speech that was presented more slowly. Based on the previous studies, our second hypothesis (H2) was that listening effort and speech intelligibility are influenced by speech rate: faster speech leads to higher listening effort (quantified by effort rating and pupillometry) and higher speech reception thresholds (SRTs).

The influence of linguistic processing on neural entrainment is comprehensively reviewed by Kösem and van Wassenhove (2017). Linguistic processing may be reflected in high oscillatory activity. The neural tracking considers the low-level fluctuations in the EEG. The question remains, if low-frequency oscillations also capture linguistic processing. Zoefel and VanRullen (2015) investigated the importance of low-level acoustic features, such as amplitude and spectral content, and higher-level features of speech, such as phoneme and syllable onsets, for neural entrainment to speech. They created three types of stimuli that covered different features and demonstrated that neural entrainment occurs to speech sounds even without fluctuations in low-level features. Thus, entrainment reflects the synchronization not only to fluctuations in low-level acoustic features but also to higher-level speech features indicating that the brain builds temporal predictions about upcoming events (Ding et al., 2017; Kösem et al., 2018). Furthermore, they showed that linguistic information is not required for neural entrainment: unintelligible speech also entrains neural oscillations and this entrainment is not enhanced by linguistic information (Millman et al., 2015; Zoefel and VanRullen, 2015). Based on these findings, we didn’t expect linguistic complexity to influence neural tracking. To evaluate this expectation we tested the hypothesis (H3) that neural tracking is affected by linguistic complexity.

The neural synchrony to time-compressed speech was investigated by Ahissar et al. (2001). They reported that a decrease in speech intelligibility produced by time-compression is accompanied by a lower synchrony between the neural response and the speech signal. This result was later confirmed by Nourski et al. (2009), who found low-frequency phase-locking only for intelligible speech rates. These studies demonstrate that a reduction in neural synchrony is related to speech comprehension using different speech rates. Other studies confirmed the finding, that improved speech intelligibility is associated with stronger neural entrainment (e.g., Gross et al., 2013; Peelle et al., 2013; for a review see Kösem and van Wassenhove, 2017) whereas others reported contradictory results; no influence of intelligibility on neural entrainment (Millman et al., 2015; Zoefel and VanRullen, 2015). The high speech rate used in the current study significantly reduced speech intelligibility compared to low and normal speech rate. Based on the findings of Ahissar et al. (2001) our fourth hypothesis (H4) was that the neural response is influenced by speech rate: faster speech leads to a reduced neural response.

An essential advantage of varying linguistic complexity and speech rate is the opportunity to create varying task demands at constant SNR. This allowed us to investigate whether neural speech tracking is only sensitive to variations in SNR and attention, as shown by Petersen et al. (2017), or whether listening effort as modulated by linguistic complexity and speech rate is also reflected in the amplitude of neural tracking. In order to investigate this research question, we correlated the individual neural tracking with both measures of listening effort; maximum pupil dilation and subjective ratings of effort. Since we hypothesized that speech rate influences both measures of listening effort as well as neural tracking, our fifth hypothesis (H5) was that there is a relation between neural tracking of speech and listening effort as quantified by effort rating and pupillometry.

## Materials and Methods

### Participants

Twenty normal-hearing participants took part in the study: 10 male and 10 female, average age of 25 years, ranging from 19 to 35 years. All participants were native German speakers, were right-handed, and reported normal vision and no history of neurological, psychiatric, or psychological disorders. The hearing thresholds of all participants were verified to be below 20 dB at the standard audiometric frequencies of 0.125, 0.25, 0.5, 0.75, 1, 1.5, 2, 3, 4, 6, and 8 kHz. One participant was excluded from the final evaluation due to poor response accuracies of the picture-matching paradigm. The exclusion criterion was an accuracy below chance level, thus below 50% accuracy. Participants were paid for their participation and informed that they could terminate their participation at any time. The study was approved by the local ethics committee of the University of Oldenburg.

### Stimuli and Tasks

#### Speech Material

The Oldenburg Linguistically and Audiologically Controlled Sentences (OLACS, Uslar et al., 2013) were used as auditory speech material. OLACS consist of seven sentence structures with different linguistic complexities. In this study we used three structures: subject-verb-object (SVO), object-verb-subject (OVS), and ambiguous object-verb-subject (ambOVS) sentences (Uslar et al., 2013). Note that OVS and ambOVS are grammatically possible in the German language, but not in many other languages, such as English. The sentences describe two entities: one is performing an action (agent) and the other is affected by that action (patient). SVO sentences are considered to be syntactically easier than OVS and ambOVS sentences since SVO sentences represent a canonical word order and are unambiguous. OVS sentences are more complex due to their non-canonical word order: the object precedes the subject. OVS sentences are unambiguous as well, whereas ambOVS sentences are ambiguous. The word that disambiguates the sentence (enables assignment of agent and patient roles) is the first noun for SVO and OVS structures, and the article of the second noun for the ambOVS structure; this word is denoted as point of target disambiguation (PTD, see Table 1).

**Table 1:** The subject-verb-object (SVO), object-verb-subject (OVS), and ambiguous object-verb-subject (ambOVS) sentence structures of the Oldenburg Linguistically and Audiologically Controlled Sentences (OLACS).

SVO	Der kluge Zauberer filmt den braven Postboten.
	The_nom_ wise_nom_ wizard_mal_ films the_acc_ honest_acc_ mailman_mal_.
OVS	Den braven Postboten filmt der kluge Zauberer.
	The_acc_ honest_acc_ mailman_mal_ films the_nom_ wise_nom_ wizard_mal_.
ambOVS	Die nasse Ente tadelt der treue Hund.
	The_amb_ wet_amb_ duck_fem_ reprimands the_nom_ loyal_nom_ dog_mal_.


**Relevant case markings are indicated by nom (nominative), acc (accusative), and amb (ambiguous case). The gender of the entities is indicated by mal (male) and fem (female). The point of target disambiguation (PTD), the point in time at which an assignment of agent and patient is possible, is marked by underlined words*.*

In order further to vary task demands, we time-expanded and time-compressed sentences of the OLACS corpus to a 25% slower and a 25% faster version. To do this, we used the pitch-synchronous overlap-add (PSOLA) procedure implemented in Praat (Boersma and van Heuven, 2001), which modifies the duration of sentences. First, PSOLA divides the speech waveform into overlapping segments and finally adds or deletes segments to achieve an extended or compressed version of the stimulus. Schlueter et al. (2014) compared different algorithms for the creation of time-compressed speech and found that the PSOLA algorithm did not produce audible artifacts to the original speech. In the following we refer to the different speech rate conditions as normal (original OLACS), slow (time-expanded OLACS), and fast (time-compressed OLACS). The original OLACS used in this study have a speech rate of 243 ± 24 syllables per minute (Uslar et al., 2013). Thus, a 25% lower speech rate results in 182 syllables per minute and a 25% higher speech rate results in 304 syllables per minute. The average length is 3.68 ± 0.28 s for sentences with a low speech rate and is 2.23 ± 0.23 s for sentences with a high speech rate.

#### Visual Material

**FIGURE 1:**
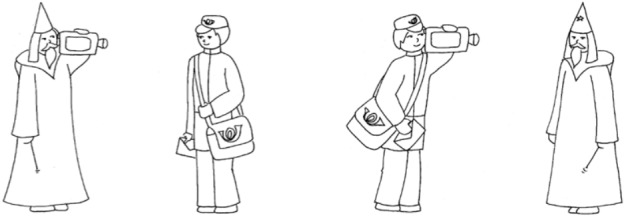
Example pictures for the OLACS sentence: “The_nom_ wise_nom_ wizard_mal_ films the_acc_ honest_acc_ mailman_mal_”. The target picture is shown on the **(left)** panel. The competing picture is shown on the **(right)** panel for the SVO sentence. Only one picture is presented per trial during the picture-matching paradigm (see Section “Picture-Matching Paradigm”).

Sentences of the OLACS corpus were presented acoustically after the visual presentation of either a target or competitor picture (see Figure 1) during the picture-matching paradigm (see “Picture-Matching Paradigm” section). The target picture shows the entities and the action as described by the sentence, whereas the competitor picture shows the same entities and action but with interchanged agent and patient roles. The development and evaluation of the OLACS pictures are described by Wendt et al. (2014).

#### Speech Recognition Measurements

The individual speech reception threshold for 80% (SRT80) word recognition for the OLACS was determined at the first session for normal, slow, and fast sentences. OLACS (female voice) were presented with a single talker masker (male voice) and the participants’ task was to repeat the sentence, spoken by the female voice, as accurately as possible. Random sequences of concatenated sentences of the Oldenburg sentence test (OLSA) presented at original speech rate were used as the single talker masker. OLSA sentences consists of five words (name, verb, number, object, and noun) and clearly differ from OLACS sentences.

Measurements started at an SNR of -5 dB and were adaptively adjusted according to the number of correctly repeated words using an adaptive level adjustment procedure. The presentation level of the subsequent sentence was calculated by

$$\Delta L=\frac{f(i)*\left(prev-tar\right)}{slope}$$

where *tar* denotes the target discrimination value, *prev* denotes the discrimination value obtained in the previous sentence, and the slope was set to 15% per dB (Brand and Kollmeier, 2002; described as A1). Participants carried out 4 blocks of OLACS with 60 sentences each (20 SVO, 20 OVS, and 20 ambOVS in random order). In the first training block, participants were familiarized with the procedure and with the sentence structures presented in the single talker masker. After this training, one block of each speech rate (normal, slow, and fast) was measured to determine the individual SRT80 values. The SNRs of the last five trials were averaged to obtain the final SRT for each sentence structure individually. The final SRT80s measured with the normal speech rate were averaged over sentence structures and used in the picture-matching paradigm as individual SRT80 across sentence structures and speech rates (see “Picture-Matching Paradigm” section).

#### Picture-Matching Paradigm

**FIGURE 2:**
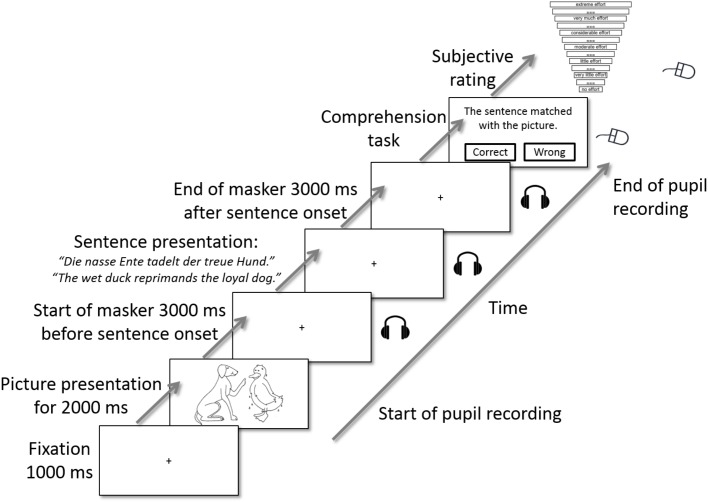
One trial of the picture-matching paradigm [modified from Wendt et al. (2016)]. A picture was displayed visually on the screen. After the picture disappeared, the single talker masker was presented acoustically and a fixation cross was displayed visually. 3 s after masker onset, a sentence of the OLACS corpus was presented acoustically. After sentence offset, the masker remained for 3 further seconds. The sound presentation ended and the participants’ task was to identify whether picture and target sentence matched. In the last step, they rated their perceived effort for this trial on a categorical scale. The categorical scale was modified from Krueger et al. (2017).

The audio–visual picture-matching paradigm used by Wendt et al. (2016) was conducted in the second session in this study. One trial of the picture-matching paradigm is illustrated in Figure 2. Each trial started with a silent baseline of 1 s while a fixation cross was displayed at the center of the screen. Afterwards, a picture with two entities (see “Visual Material” section) was displayed for 2 s. The picture disappeared, the fixation cross was displayed again, and the acoustic presentation of the single talker masker started. The competing talker was presented alone for 3 s, and then the OLACS sentence was presented in addition. 3 s after sentence offset, the single talker masker stopped, and the participants’ task was to match the visually displayed picture with the acoustically presented OLACS, while ignoring the competing talker. To indicate their decision, participants pressed the right or left button on the computer mouse. In the last step, participants rated their perceived effort for that trial on a categorical scale ranging from “no effort” to “extreme effort” (Krueger et al., 2017). The scale was slightly modified by removing the top category, which normally occurs when the stimulus is noise only; since our stimuli were presented at a high SNR, a condition with only noise never occurred in our experiment. The fixation cross was displayed during sound presentation in order to reduce the occurrence of disturbing eye movements.

The demand level of the task was varied using two parameters (described in detail in the “Speech Material” section):

(1) The level of linguistic complexity was varied using three different sentence structures of the OLACS corpus (SVO, OVS, and ambOVS).
(2) The speech rate was varied using versions of the original speech material that were 25% slower and 25% faster.

In total, 200 trials were performed during the picture-matching paradigm: 30 of each of the six parameter combinations (level of linguistic complexity × speech rate) and 20 filler trials, where the figures or the action displayed on the picture did not match the sentence, to keep the participants’ attention on the task. The filler trials were not analyzed. The amount of “yes/match” and “no/no match” trials are equal in all conditions. Approximately 28% of the OLACS sentences were repeated in the picture-matching paradigm after using them in the SRT80 procedure. The response accuracies during the picture matching paradigm were very high for 19 of 20 participants. One participant was excluded from the data analysis since the response accuracies were below 50%. Averaged across all participants, the highest response accuracies were found for the SVO sentence structure (slow: 92.6 ± 4.8%, fast: 91.4 ± 5.6%), followed by the OVS sentence structure (slow: 91.9 ± 4.6%, fast: 88.4 ± 5.8%) and the ambOVS sentence structure (slow: 91.2 ± 4.5%, fast: 87.7 ± 8.1%). Even though linguistically more complex sentences and faster presented speech produced numerically lower response accuracies, statistical analysis revealed no significant difference in response accuracies [χ^2^(5) = 8.65, *p* > 0.05].

### Verbal Working Memory

At the end of the first session, participants performed the German version of the reading span test (RST; Carroll et al., 2015). The test determines the individuals’ verbal working memory capacity (WMC). WMC reflects the cognitive abilities of a listener when managing the processing of information (Besser et al., 2013). Moreover, WMC is related to speech recognition and to compensations of demands (Wendt et al., 2016). In the current study, WMC was measured to relate differences in cognitive abilities to measures of speech reception and listening effort. Therefore, individual WMC scores were correlated with SRT, PPD, and ESCU. The RST consists of 54 sentences with 4 to 5 words which were presented visually in short segments on a screen. Participants were instructed to read out loud what was displayed and to memorize the sentences. Furthermore, after each sentence, they had to judge, within 1.75 s, whether the sentence was plausible or not. After 3 to 6 sentences (randomized selection) they were asked to recall the first or the last word of the sentences. The score of the RST is the percentage of correctly recalled words across all 54 sentences. In the first training block, consisting of three sentences, participants became familiar with the task.

### Apparatus

Measurements took place in a sound-isolated booth where the participants were seated comfortably on a chair in front of a monitor. The acoustical and visual presentations were controlled via Matlab (Mathworks Inc., Natick, MA, United States) and the Psychophysics Toolbox (PTB, Version 3; Brainard, 1997). The acoustic signals were forwarded from the RME sound card (Audio AG, Haimhausen, Germany) to ER2 insert earphones (Etymotic Research Inc., Elk Grove Village, IL, United States). The visually presented stimuli were displayed on a 22″ computer monitor with a resolution of 1920 pixels × 1080 pixels. Pupillometry was conducted during the picture-matching paradigm with the EyeLink1000 desktop mount eye-tracker (SR Research Ltd., Mississauga, Canada) with a sampling rate of 500 Hz in remote configuration (without head stabilization). A nine-point fixation calibration at the start of the recording was completed. The illumination in the booth was kept constant for all participants. EEG was recorded using the Biosemi ActiveTwo system (BioSemi, Amsterdam, Netherlands) from an elastic cap with 64 active electrodes positioned at 10–20 system locations and two electrodes placed on the right and left mastoids. One additional electrode was placed below the right eye to register eye blinks. The impedances were kept below 20 kΩ. EEG data were recorded with a sampling frequency of 512 Hz and filtered during acquisition applying an online high pass filter at 0.16 Hz and a low pass filter at 100 Hz.

### Data Analysis and Statistical Analysis

#### EEG Data Processing and Calculation of Neural Speech Tracking

The EEG data processing and the extraction of speech-onset envelopes (SOEs) described in the following are similar to Petersen et al. (2017). The EEG data were analyzed using customized MATLAB (Mathworks Inc., Natick, MA, United States) scripts, the EEGLAB toolbox (Delorme and Makeig, 2004), and the FieldTrip toolbox (Maris and Oostenveld, 2007). First, the raw EEG data were re-referenced to the mean of the electrodes placed on the left and right mastoids. Second, independent component analysis (ICA) was applied to identify eye blinks and lateral eye movements, which were then removed from the EEG data of each participant using the EEGLAB plug-in CORRMAP (Viola et al., 2009). The data were band-pass filtered from 0.5 to 45 Hz, down-sampled to 250 Hz, and epoched from 6 before to 6 s after sentence onset.

Speech-onset envelopes (SOEs) were extracted from each sentence presented in the picture-matching paradigm. To achieve this, the absolute of the Hilbert transform was low-pass filtered with a 3rd-order Butterworth filter with a cut-off frequency of 25 Hz. Afterwards, the first derivative was taken from the filtered signal. In the last step, it was half-wave rectified, the negative was half clipped, and the resulting signal was down-sampled to a sampling rate of 250 Hz.

The neural tracking of speech is the phase-locked neural response to the SOE of the corresponding sentences. Therefore, neural tracking of speech was measured by calculating the cross-correlation between the processed EEG epoch from sentence onset until offset and the SOE of the corresponding sentence for all 200 trials. The first 200 ms were omitted from the analysis in order to avoid the strong influence of the onset response.

Statistical comparisons between the neural tracking of speech for the sentence structures (SVO, OVS, and ambOVS) and speech rates (slow and fast) were calculated using the cluster-based permutation procedure implemented in the FieldTrip toolbox (Maris and Oostenveld, 2007). First, this procedure calculated dependent samples *t*-statistics between cross-correlations of respective conditions (e.g., slow vs. fast, collapsed over sentence structures) for each time sample and channel. Time samples with *t*-statistics of *p* < 0.05 and with at least two neighboring channels with *t*-statistics of *p* < 0.05 were constructed to connected clusters. In the second step, the procedure calculated the cluster-level statistics by taking the sum of *t*-values within each cluster. To correct for multiple comparisons, the cluster-level statistic was then compared to a reference distribution. The reference distribution was obtained by randomly permuting trials of the conditions and calculating the maximum of the summed *t*-values for 1000 iterations. If the summed *t*-values of the identified cluster exceeded the 95% percentile (*p* < 0.025, two-sided) of the permutation distribution, the cluster was considered significant (for more details, see Maris and Oostenveld, 2007).

#### Pupil Data Analysis

The pupil data analysis described in the following is similar to the analysis performed by Wendt et al. (2016). Pupil data were first cleaned by removing eye blinks: samples that were more than three standard deviations below the average pupil dilation were classified as eye blinks and removed from the data. The deleted samples were linearly interpolated from 350 ms before to 700 ms after the eye blink (Wendt et al., 2016). Trials that required 20% or more interpolation were completely removed from further analysis. Afterwards, a four-point moving average filter with a symmetric rectangular window was used to smooth the de-blinked trials and to remove any high-frequency artifacts. Finally, data were normalized by subtracting the average of the last second before sentence presentation as baseline from the data. Differences in the individual peak-pupil dilations (PPDs) were statistically analyzed using a repeated-measures analysis of variance (ANOVA) with linguistic complexity and speech rate as within-subject factors.

## Results

### Speech Reception Thresholds (SRTs)

**FIGURE 3:**
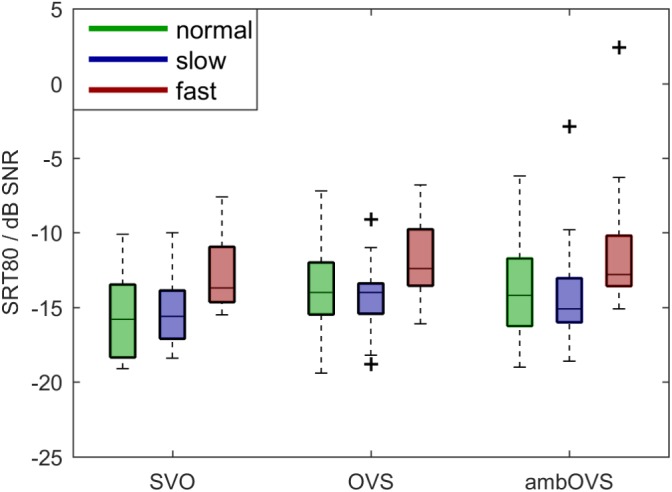
Boxplots of participants’ SRT80s for normal (green), slow (blue), and fast (red) speech rates and SVO, OVS, and ambOVS sentence structures. Repeated measures ANOVA revealed a main effect of linguistic complexity with significant lower SRT80s for SVO sentences compared to OVS and ambOVS sentences and an effect of speech rate with higher SRT80s for fast speech compared to normal and slow speech.

To investigate the influence of linguistic complexity and speech rate on speech intelligibility (H1 and H2), SRT80s were measured for OLACS presented at two different speech rates. Figure 3 shows boxplots of participants’ SRT80s. The horizontal line inside the box represents the median, bottom and top edges of the box represent the 25th and 75th percentiles (interquartile range, IQR). The whiskers of the box are the maximum and minimum values within 1.5 ^∗^ IQR. Outliers outside the range of the whiskers are indicated with a “+” symbol. The SRT80s were statistically analyzed using a repeated-measures analysis of variance (ANOVA) with linguistic complexity and speech rate as within-subject factors. The statistical analysis revealed a main effect of linguistic complexity [*F*(2,36) = 6.97, *p* = 0.003, $\eta_{p}^{2}$ = 0.279] and speech rate [*F*(2,36) = 15.002, *p* < 0.001, $\eta_{p}^{2}$ = 0.455]. No interaction effect between linguistic complexity and speech rate was found. The Bonferroni corrected *t*-test as *post hoc* analysis showed that the SRT80 of the SVO sentence structure was significantly lower than the SRT80 of the OVS (*p* = 0.03, mean difference -1.26, 95%-CI [-2.41, -0.12]) and ambOVS (*p* = 0.02, mean difference -1.51, 95%-CI [-2.83, -0.195]) sentence structure. Furthermore, the SRT80 was significantly higher for fast speech than for normal speech (*p* = 0.001, mean difference 2.395, 95%-CI [0.959, 3.83]) and slow speech (*p* = 0.001, mean difference 2.51, 95%-CI [0.984, 4.04]).

### Subjectively Rated Listening Effort of the Picture-Matching Paradigm

**FIGURE 4:**
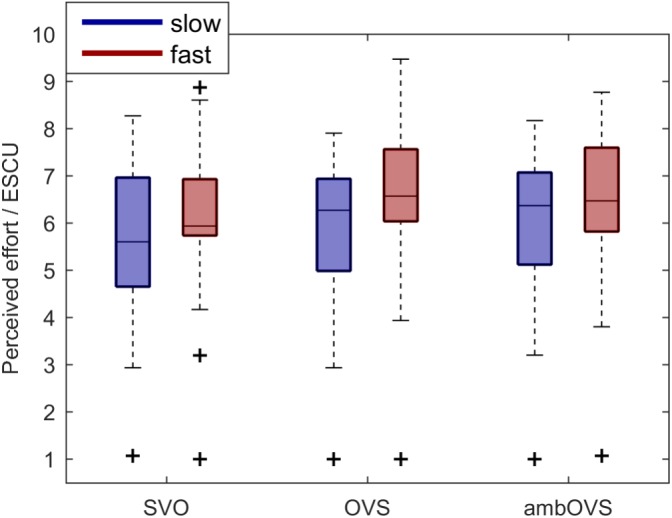
Boxplots of participants’ perceived effort for the picture-matching paradigm for the slow (blue) and fast (red) speech and the SVO, OVS, and ambOVS sentence structures. Repeated measures ANOVA revealed a main effect of linguistic complexity with significant lower effort ratings for SVO sentences compared to OVS and ambOVS sentences and an effect of speech rate with higher effort ratings for fast speech compared to slow speech.

To investigate the influence of linguistic complexity and speech rate on listening effort (H1 and H2), participants rated their perceived effort on a rating scale for OLACS presented at two different speech rates. Figure 4 shows boxplots of participants’ perceived listening effort in effort scale categorical units (ESCUs) for the picture-matching paradigm. Fast speech resulted in the highest median perceived effort for all sentence structures (SVO: 5.9 ESCU, OVS: 6.57 ESCU, ambOVS: 6.47 ESCU) in comparison to slow speech (SVO: 5.6 ESCU, OVS: 6.27 ESCU, ambOVS: 6.37 ESCU). The effort ratings were statistically analyzed using a repeated-measures analysis of variance (ANOVA) with linguistic complexity and speech rate as within-subject factors. Statistical analysis revealed a main effect of linguistic complexity [*F*(2,36) = 7.55, *p* = 0.002, $\eta_{p}^{2}$ = 0.296] and speech rate [*F*(1,18) = 17.13, *p* = 0.001, $\eta_{p}^{2}$ = 0.488] with higher effort ratings for fast sentences. No interaction effect between linguistic complexity and speech rate was found. The *post hoc* analysis revealed that the subjectively rated effort was lower for the SVO sentence structure compared to the OVS (*p* = 0.007, mean difference -0.325, 95%-CI [-0.57, -0.08]) and the ambOVS (*p* = 0.008, mean difference -0.353, 95%-CI [-0.618, -0.087]) sentence structure.

### Pupil Dilation

**FIGURE 5:**
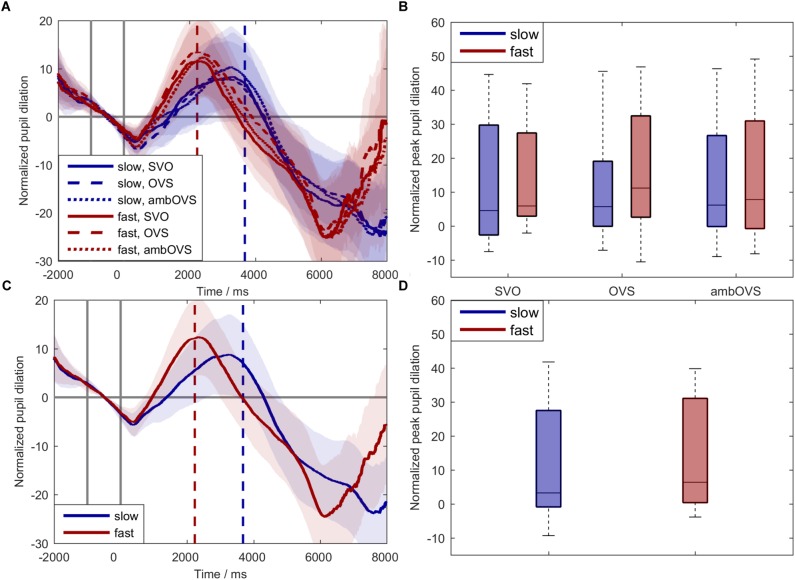
Normalized pupil dilations across participants. Blue: slow speech; red: fast speech; solid lines: SVO; dashed lines: OVS; dotted lines: ambOVS. **(A)** Shows the normalized pupil dilation over time with the corresponding 95% confidence interval. **(B)** Shows boxplots of peak-pupil dilations (PPDs). **(C)** Shows the normalized pupil dilation over time with the corresponding 95% confidence interval collapsed across sentence structures. **(D)** Shows boxplots of PPDs collapsed across sentence structures. The first two solid vertical lines in **(A,C)** indicate the range of the baseline used for normalization. The blue and red dashed vertical lines indicate the averaged ends of slow and fast sentences. Repeated measures ANOVA revealed no main effect of linguistic complexity but a main effect of speech rate with larger PPDs for fast speech compared to slow speech.

To investigate the influence of linguistic complexity and speech rate on listening effort (H1 and H2), participant’s pupil dilations were recorded during the audio-visual paradigm. Figure 5 shows averages and boxplots of participants’ pupil dilation. Figure 5A shows the averaged normalized pupil dilation over time with the corresponding 95% confidence interval for the six conditions (speech rate × linguistic complexity). For statistically analyzing the influence of speech rate and linguistic complexity on listening effort based on pupil dilations we analyzed the individual peak-pupil dilations (PPDs, Figure 5B). The statistical analysis revealed a main effect of speech rate on PPDs [*F*(1,18) = 15.831, *p* = 0.001, $\eta_{p}^{2}$ = 0.468] with higher PPDs for fast speech. No effect of linguistic complexity on the PPDs [*F*(2,36) = 0.22, *p* = 0.8, $\eta_{p}^{2}$ = 0.012] and no interaction effect between linguistic complexity and speech rate was observed. Since linguistic complexity did not affect pupil dilation, we collapsed the data across sentence structures for a better visualization (Figure 5C,D).

### Neural Tracking of Speech

**FIGURE 6:**
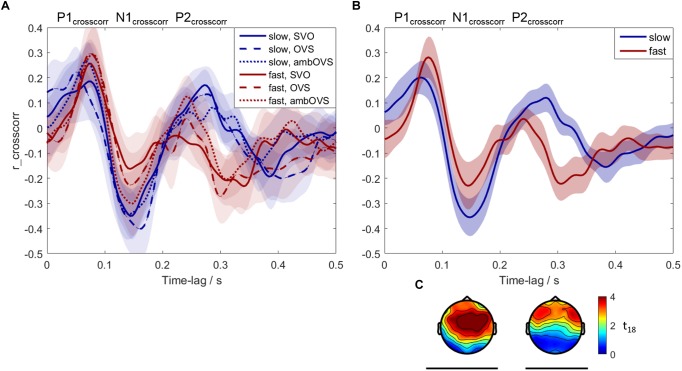
Neural tracking of speech averaged across participants. Blue: slow speech; red: fast speech; solid lines: SVO; dashed lines: OVS; dotted lines: ambOVS. **(A)** Shows the averaged cross-correlations with the 95% confidence intervals between the speech-onset envelopes and the EEG signal. **(B)** Shows the cross-correlations with the corresponding 95% confidence intervals collapsed across sentence structures. The results in **(A,B)** were averaged over 19 participants and averaged over the 45 electrodes which were common for both significant clusters. The three components P1_crosscorr_, N1_crosscorr_, and P2_crosscorr_ are marked. **(C)** Shows the topographical plots of *t*-values for the two significant clusters calculated with the cluster-based permutation procedure. Time lags at which the slow and fast speech differed significantly are indicated by black lines below the corresponding time intervals and topographical plots.

To investigate the influence of linguistic complexity and speech rate on neural tracking of speech (H3 and H4), neural tracking was measured based on the recorded EEG. Figure 6 shows neural speech tracking for the three sentence structures (SVO, OVS, and ambOVS) and for slow and fast speech. Figure 6A shows the cross-correlations between EEG and SOE for the six conditions (speech rate × linguistic complexity). Three components, denoted as P1_crosscorr_, N1_crosscorr_, and P2_crosscorr_ are present. The cluster statistics revealed no significant difference between sentence structures. The *p*-values for the different clusters are in the range of 0.04 and 0.92 with an average of 0.6. Almost all clusters showed *p*-values of >0.2 except of the comparison of SVO and ambOVS sentence structure of the fast speech rate. Here, two clusters reached p-values of 0.04 and 0.08 (significant was reached if *p* < 0.025). Since linguistic complexity did not affect neural tracking, we focused our analysis on the influence of speech rate. Therefore, we collapsed the data across sentence structures and calculated the cross-correlations for slow and fast speech (Figure 6B,C). The cluster statistics computed between cross-correlations of slow and fast speech identified two significant time clusters where neural tracking of fast and slow speech differed significantly: a significant negative cluster N1_crosscorr_ at 0.072–0.196 s (61 electrodes, *p* < 0.001, see Figure 6C) and a positive cluster P2_crosscorr_ at 0.244–0.352 s (45 electrodes, *p* < 0.001, see Figure 6C). The difference in neural tracking between slow and fast speech at the time of the positive cluster P2_crosscorr_ may have resulted from faster processing of the faster sentences. The peak of P2_crosscorr_ for fast speech (*M* = 0.24, *SE* = 0.01) is on average earlier compared to slow speech (*M* = 0.29, *SE* = 0.01) (see Figure 6B). This difference in latency between slow and fast speech is significant [*t*(18) = 2.473, *p* = 0.024]. Thus, fast speech influences the amplitude of neural tracking as well as the processing duration. In other words, the increase of speech rate accelerates the P2_crosscorr_ occurrence which might indicate a faster P2_crosscorr_ related processing of the brain. Faster presented speech might be processed faster in order to receive all incoming information.

### Correlation Between Listening Effort and Neural Tracking of Speech

**FIGURE 7:**
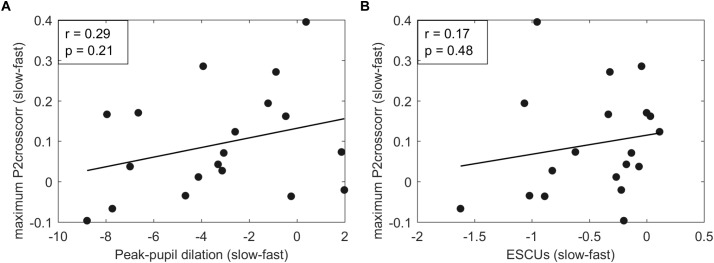
**(A)** Shows correlations between P2_crosscorr_ amplitudes of neural tracking and peak-pupil dilations (PPDs). **(B)** Shows correlations between P2_crosscorr_ amplitudes of neural tracking and subjective effort ratings in ESCUs. Pearson’s correlation revealed no correlation between PPDs and neural tracking (*r* = 0.29, *p* = 0.21) or between ESCUs and neural tracking (*r* = 0.17, *p* = 0.48).

The goal of the current study was to investigate whether the neural response is affected by listening effort as quantified by effort rating and pupillometry. Therefore, the relation between neural tracking and PPDs and perceived effort was investigated. PPDs and perceived effort in ESCUs were larger for sentences presented with a high speech rate, whereas neural tracking showed smaller amplitudes at significant time clusters. To investigate the relation of listening effort and neural speech tracking, we correlated the differences between slow and fast speech of individual PPDs and ESCUs with the individual amplitude of the P2_crosscorr_ of the neural tracking response (see Figure 7). PPDs, ESCUs, and P2_crosscorr_ amplitudes were collapsed across sentence structures. Pearson’s correlation revealed no relation between PPDs and neural tracking (*r* = 0.29, *p* = 0.21) or between ESCUs and neural tracking (*r* = 0.17, *p* = 0.48).

### Correlation Between WMC and SRT80, PPD, and ESCU

The individuals’ WMC was examined for correlations with SRT80, PPD, and ESCU for all speech rates and sentence structures. Pearson’s correlation was conducted using Bonferroni adjusted alpha levels of 0.003 per test (0.05/18). The statistical analysis revealed no significant correlations.

## Discussion

In this study, participants listened to sentences with varying degrees of linguistic complexity presented with a low or a high speech rate during a picture-matching paradigm. We investigated the impact of linguistic complexity and speech rate on listening effort, measured with pupillometry and subjectively rated on a categorical scale, and neural tracking of speech, measured with EEG. Furthermore, the relation between listening effort and neural tracking of speech was investigated.

### The Impact of Linguistic Complexity and Speech Rate on Speech Reception Thresholds (SRT80s)

Earlier studies showed that processing of sentences that are syntactically more complex results in decreased speech comprehension (Uslar et al., 2013), increased processing effort (Wendt et al., 2016), and increased processing duration (Wendt et al., 2014, 2015; Müller et al., 2016). The present study showed a systematic effect of complexity on speech intelligibility, with the lowest SRT80 for the SVO sentence structure. Statistical analysis revealed a significant difference between SVO sentence structure and OVS and ambOVS sentence structure. Uslar et al. (2013) measured SRT80 for SVO, OVS, and ambOVS sentence structures in different background noises (quiet, stationary noise, and fluctuating noise) and reported that the results were influenced by the background noise. In fluctuating noise, they reported that ambOVS sentences produced the highest SRT80, which differed statistically from SRT80 for SVO and OVS sentences. Our results are partly in line with results reported by Uslar et al. (2013) with respect to the fluctuating noise condition, since the competing talker situation in our experiment is best comparable with their fluctuating noise condition. In the present study, recognition performance of the ambOVS structure differed from SVO but not that of OVS sentence structure. The slightly different results between studies can be explained by very small differences in SRT80 between sentence structures that are in the order of 1–2 dB and by the different background noises. Uslar et al. (2013) discussed that the strong difference between the fluctuating listening condition and two others (quiet and stationary noise) is presumably related to the ability to listen into the gaps of a modulated noise masker, which improves speech intelligibility for listeners with normal hearing (e.g., Festen and Plomp, 1990; Bronkhorst, 2000 for a review). They expected the effect of linguistic complexity on speech intelligibility to be more pronounced for a single talker masker. This expectation could not be confirmed by our results.

Speech rate had a clear effect on speech intelligibility, with higher SRT80s for fast speech than for normal and slow speech. This result is in line with other studies that showed decreasing speech comprehension with increasing speech rate (e.g., Versfeld and Dreschler, 2002; Liu and Zeng, 2006; Schlueter et al., 2014).

### Verbal Working Memory Capacity (WMC) and Correlations With SRT80, PPD, and ESCU

In this study WMC was determined with the German reading span test (Carroll et al., 2015) and examined for correlations with SRT80, PPD, and ESCU for individual participants. The listeners’ cognitive ability is associated with speech in noise performance in hearing impaired and normal-hearing listeners (Dryden et al., 2017). However, no significant correlations between WMC and SRT80, PPD, and ESCU were found. Those result support the findings of Füllgrabe and Rosen (2016b), that WMC might not be a good predictor of speech in noise scores in younger normal-hearing listeners. They reported that WMC, measured with the reading span test, predicts less than 2% of the variance in speech in noise intelligibility for young normal-hearing listeners. However, higher correlations between WMC and speech in noise scores were found for older listeners (e.g., Füllgrabe and Rosen, 2016a).

According to previous literature, better cognitive abilities, such as higher WMC, are associated with listening effort, as indicated by pupil size (Zekveld et al., 2011; Wendt et al., 2016). For example, Wendt et al. (2016) reported significant correlations between WMC, as indicated with digit span scores, and listening effort. However, those correlations were only revealed for less complex sentence structures. Wendt and colleagues argued that cognitive resources may be exhausted for complex situations, which might explain the missing correlations for more complex situations (Johnsrude and Rodd, 2016). In contrast to previous studies, no significant correlations between WMC and listening effort were found in the current study.

### The Impact of Linguistic Complexity and Speech Rate on Listening Effort

The impact of linguistic complexity and speech rate on listening effort (H1 and H2) was investigated based on subjectively rated effort (perceived effort) and pupil dilation. The ratings of perceived effort showed that the SVO sentence structure was rated as least effortful. This result is in line with other studies that reported larger perceived effort for more complex sentence structures (Wendt et al., 2016). Thus, the SVO sentence structure, considered to be the easiest because of its word order and its common use in the German language (Bader and Meng, 1999), produced the lowest speech comprehension thresholds and resulted in the lowest perceived effort.

In contrast to previous studies, we did not observe an influence of linguistic complexity on pupil dilation (Piquado et al., 2010; Wendt et al., 2016). Wendt et al. (2016), who used the corresponding speech material in the Danish language, showed a clear influence of linguistic complexity on pupil dilation, with increasing complexity resulting in lager pupil dilations. As shown by earlier studies, linguistic complexity had a strong influence on processing duration (Wendt et al., 2014, 2015; Müller et al., 2016) and participants needed more time to process more complex sentence structures. We also expected to find such differences in processing duration in the development of the pupil dilation. One reason for the missing effect in our data might be the influence of speech rate on pupil dilation.

Speech rate showed a significant influence on perceived effort. Fast speech produced the highest SRT and was rated as most effortful. Our results are in line with other studies that showed a relation between perceived effort and SNR (Rudner et al., 2012; Wendt et al., 2016). Nevertheless, differences in ratings among sentence structures and speech rates were rather small, with effects lower than 1 ESCU.

Looking closely at Figure 4, it turns out that one participant produced outliers at an ESCU of one. This participant rated every situation as “no effort,” independent of sentence structure and speech rate. This may have resulted from low motivation; Picou and Ricketts (2014) demonstrated that perceived effort could be affected by the listeners’ motivation. Furthermore, the framework for understanding effortful listening (FUEL), introduced by Pichora-Fuller et al. (2016), nicely demonstrates the relation between motivation, demands, and effort. They suggest reduced motivation when demands are constant, resulting in decreased effort. However, the exclusion of the participant that produced the outliers from the statistical analysis did not change the conclusion of the statistical outcome.

The results of pupil dilations are in line with the subjective effort ratings regarding speech rate. The pupil dilations showed a significant difference between slow and fast speech: fast speech resulted in larger pupil dilations. Moreover, the visual inspection shows not only a faster but also a steeper development of pupil size. This strong influence of speech rate seemed to dominate the development of pupil size and may have eliminated the effect of linguistic complexity.

Taken together, the impact of linguistic complexity and speech rate on listening effort was not consistent between subjectively rated effort and effort measured with pupil dilation. Linguistic complexity had an effect on perceived effort but not on pupil dilations. Our hypothesis (H1), that the amount of listening effort is influenced by the level of linguistic complexity, with higher complexity leading to higher listening effort, was confirmed by the results of the current study for perceived effort but not for pupil dilations. Differences between results measured with subjectively rated effort and with pupil dilations were also demonstrated by Wendt et al. (2016). Our hypothesis (H2), that the amount of listening effort is influenced by speech rate, with faster speech leading to higher listening effort, was also confirmed.

### The Impact of Linguistic Complexity and Speech Rate on Neural Tracking of Speech

The impact of linguistic complexity and speech rate on neural tracking of speech (H3 and H4) was investigated based on EEG recordings using the data analysis introduced by Petersen et al. (2017). The time course of the neural tracking of attended speech measured in our study is comparable with earlier studies (Ding and Simon, 2012; Horton et al., 2013; Kong et al., 2014; O’Sullivan et al., 2014; Zoefel and VanRullen, 2015; Petersen et al., 2017) with a positive deflection at around 80 ms, denoted as P1_crosscorr_, a negative deflection at around 150 ms, denoted as N1_crosscorr_, and a second positive deflection at around 260 ms, denoted as P2_crosscorr_. These denotations were adapted from Petersen et al. (2017). The studies mentioned above investigated differences in neural tracking between attended and ignored speech and reported an attentional effect at N1_crosscorr_ at around 150 ms with a reduced amplitude of neural tracking for the ignored stimulus. However, Ding and Simon (2012) showed earlier effects at around 100 ms and Petersen et al. (2017) reported effects up to 200 ms. These variations may have arisen from different groups of listeners with normal hearing and with impaired hearing (Petersen et al., 2017). Our study investigated the influence of linguistic complexity and speech rate on neural tracking. We did not observe an influence of linguistic complexity on neural tracking of speech. No significant differences in the amplitude of neural tracking were identified between sentence structures. Many studies investigated whether phase-locking to the speech envelope reflects the synchronization to acoustical features of the speech stimulus and/or the synchronization to phonetic and linguistic features. Some studies reported an influence of intelligibility on the amplitude of neural tracking, with a stronger representation for intelligible speech compared to unintelligible speech (e.g., Luo and Poeppel, 2007; Kerlin et al., 2010; Peelle et al., 2013). However, other studies reported contradictory results suggesting that entrainment is not driven by linguistic features (e.g., Howard and Poeppel, 2010; Millman et al., 2015; Zoefel and VanRullen, 2015; Baltzell et al., 2017). Since sentences of our speech material only differ in their linguistic features, our results are in line with the aforementioned studies suggesting that linguistic features do not influence the neural tracking.

Speech rate had a strong influence on neural tracking: first, the amplitude of neural tracking was reduced for fast speech, and second, the neural tracking was delayed for slow speech. Different studies investigated the neural phase-locking for time-compressed speech (Ahissar et al., 2001; Nourski et al., 2009; Hertrich et al., 2012). Ahissar et al. (2001) showed correlations between phase-locking and comprehension for different time-compression ratios. Nourski et al. (2009) confirmed the results with lower temporal synchrony for compression ratios that resulted in unintelligible speech and noted that time-compressed sentences are also reduced in duration, which might elicit large neural onset responses that disturbed the phase-locking. They compressed sentence durations up to extreme compression ratios of 0.2, leading to sentence durations of down to 0.29 s. Sentences in our experiment were reduced/expanded only moderately to 25% of their original rate, which leads to a minimum duration of 1.79 s and a maximum duration 4.66 s. Thus, the influence of the neural onset response on phase-locking as reported by Nourski et al. (2009) was reduced in our experiment. Furthermore, to avoid the influence of the neural onset response on the correlation, we excluded the first 200 ms of the sentences and the corresponding EEG from the correlation analysis, as done by Aiken and Picton (2008) and Horton et al. (2013), for example. Hertrich et al. (2012) cross-correlated magnetoencephalography (MEG) recordings and speech envelopes of moderately fast and ultrafast speech and found a reduction for unintelligible ultrafast speech in the M100, which is the magnetic counterpart of the electrical N100 or N1. The aforementioned studies found an influence of speech rate on neural tracking with reduced neural tracking for time-compressed and unintelligible speech. Even though sentences presented with a high speech rate in our experiment are still intelligible, we also found a reduction in neural tracking at a time-lag of the significant negative cluster at N1_crosscorr_ at around 150 ms. These results indicate that the cross-correlation at around 100–150 ms is not only influenced by the SNR or the participants attention (as shown by Petersen et al., 2017) but also by other stimulus properties and/or cognitive factors that are influenced by speech rate. A further significant difference that we found between slow and fast speech was at the positive cluster P2_crosscorr_ at around 250–300 ms. Here we also found a significant reduction in amplitude of the cross-correlation for fast speech. Only some of the studies that measured neural tracking based on cross-correlations could observe a P2_crosscorr_ component and suggested that its development depends on task difficulty (Horton et al., 2013; Petersen et al., 2017). Here the combination of linguistic complexity and speech rate in order to vary task demands may have increased task difficulty so that a P2_crosscorr_ was elicited. Interestingly, the differences in P2_crosscorr_ between slow and fast speech occurred not only for amplitude but also for timing. The P2_crosscorr_ of fast speech appeared earlier than the P2_crosscorr_ of slow speech. A difference in P2_crosscorr_ timing was observed before for attended versus ignored speech. Petersen et al. (2017) measured cross-correlations for attended and ignored speech and found earlier N1_crosscorr_ and P2_crosscorr_ for the ignored condition. Since Petersen et al. (2017) did not analyze this difference in timing, it remains unclear whether this effect is caused by speech rate. It is very important to note that in our study only the timing of the P2_crosscorr_ was affected by speech rate. The appearance of P1_crosscorr_ and N1_crosscorr_ were not affected. To the authors’ knowledge, this adaptation hasn’t been observed before in other studies.

Taken together, the different sentence structures did not influence neural tracking of speech, which rejects our hypothesis (H3) that neural tracking is affected by linguistic complexity. However, speech rate affected neural tracking with a stronger neural tracking for slow speech, which confirms our hypothesis (H4) that neural speech tracking is influenced by speech rate, i.e., faster speech leads to a weaker neural tracking. Interestingly, not only the amplitude but also the timing of the neural tracking was influenced by speech rate. Fast speech was also processed faster, which indicates that the processing adapts to the auditory input for an optimal stimulus processing (Bosker and Ghitza, 2018).

### Relation Between Listening Effort and Neural Tracking of Speech

The focus of attention to a specific talker in difficult listening environments may enhance effortful listening (Pichora-Fuller et al., 2016), but also enhances the neural tracking of the attended speech stream (e.g., O’Sullivan et al., 2014; Mirkovic et al., 2015; Petersen et al., 2017). Since selective attention showed an influence on neural tracking, we investigated whether listening effort caused by linguistic complexity and speech rate and quantified by subjective ratings and pupillometry is reflected on neural tracking as well (H5). Petersen et al. (2017) investigated the effect of background noise on neural tracking of attended speech and reported a reduced amplitude of neural tracking for lower SNR. Since we kept the individual SNR constant and varied auditory task demands using linguistic complexity and speech rate, we investigated whether neural speech tracking is only sensitive to variations in SNR, as shown by Petersen et al. (2017), or if listening effort as quantified by effort rating and pupillometry may explain differences in neural tracking. Petersen et al. (2017) also demonstrated that attention, which is known to enhance listening effort, modulates neural tracking. However, the selective filtering and the actual amount of effort, produced by attention, is not differentiable. Therefore, we decided to focus on further factors that modulates listening effort (ling. complexity and speech rate) and to investigate if these factors also lead to an influence on neural tracking. In this study, speech rate showed a strong influence on neural tracking and listening effort when considering results averaged across participants. Therefore we correlated the individual amplitude of neural tracking with individual results of listening effort collapsed across sentence structures to investigate the impact of effort on neural tracking. No significant correlations between the amplitude of neural tracking and subjectively rated effort and between the amplitude of neural tracking and pupil dilations were measured, even though both measures were affected by speech rate. The missing correlation might be explained by other factors that influence these physiological measures (EEG and pupil dilation) or the subjectively rated effort. For instance, the pupillary response is sensitive to arousal, as summarized by Johnsrude and Rodd (2016). Uncontrolled arousal caused by the unfamiliar laboratory situation might have influenced the pupillary response in a different way than the EEG of the participants. Furthermore, neural tracking is represented by the correlation of the speech-onset envelope of the presented speech with the corresponding EEG signal. Thus, neural tracking is strongly influenced by acoustic properties of the speech stream and cognitive factors (like attention), whereas pupillary responses and perceived effort may be more influenced by cognitive factors.

Consequently, we could not demonstrate a significant relation between the amplitude of neural tracking and listening effort as quantified by subjective effort rating and pupillometry. Therefore, our last hypothesis (H5), that there is a relation between listening effort and neural tracking of speech, is not supported.

## Conclusion

First, we demonstrated that linguistic complexity for German sentences did not affect neural tracking and listening effort measured with pupil dilations. Second, speech rate showed a strong influence on subjectively rated effort, pupil dilations, and neural tracking of speech. Interestingly, not solely the amplitude of neural tracking, but also the latency was affected by speech rate. Sentences presented with a high speech rate resulted in an earlier P2_crosscorr_. Thus, the brain adapts to the auditory input for an optimal stimulus processing. Third, we could not demonstrate a relation between neural tracking and listening effort even though both measures showed a clear influence of speech rate averaged across participants.

## Author Contributions

JM, DW, and TB formulated the research question. JM, DW, BK, SD, and TB designed the study. JM carried out the experiments. JM, DW, and TB analyzed the data and wrote the final paper.

## Conflict of Interest Statement

The authors declare that the research was conducted in the absence of any commercial or financial relationships that could be construed as a potential conflict of interest.
